# Optimism, Resilience, and General Self-Efficacy Predict Lower Somatic Burden during the COVID-19 Pandemic

**DOI:** 10.3390/healthcare12131338

**Published:** 2024-07-05

**Authors:** Alena Zolotareva, Anna Khegay, Elena Voevodina, Igor Kritsky, Roman Ibragimov, Nina Nizovskih, Vsevolod Konstantinov, Arina Malenova, Irina Belasheva, Natalia Khodyreva, Vladimir Preobrazhensky, Kristina Azanova, Lilia Sarapultseva, Almira Galimova, Inna Atamanova, Anastasia Kulik, Yulia Neyaskina, Maksim Lapshin, Marina Mamonova, Ruslan Kadyrov, Ekaterina Volkova, Viktoria Drachkova, Andrey Seryy, Natalia Kosheleva, Evgeny Osin

**Affiliations:** 1 International Laboratory of Positive Psychology of Personality and Motivation, HSE University, 101000 Moscow, Russia; akhegai@hse.ru (A.K.); eyuvoevodina@gmail.com (E.V.); vnpreobrazhenskiy@edu.hse.ru (V.P.); kaazanova@edu.hse.ru (K.A.); nathalie.74@mail.ru (N.K.); 2 Institute of Immunology and Physiology, Ural Branch of the Russian Academy of Sciences, 620049 Ekaterinburg, Russia; igor81218@gmail.com (I.K.); ibragimovroman98@yandex.ru (R.I.); 3 Department of Psychology, Vyatka State University, 610000 Kirov, Russia; nina.nizovskikh@gmail.com; 4 Department of General Psychology, Penza State University, 440026 Penza, Russia; konstantinov_vse@mail.ru; 5 Department of General and Social Psychology, Dostoevsky Omsk State University, 644077 Omsk, Russia; malyonova@mail.ru; 6 Department of General Psychology and Personality Psychology, North-Caucasus Federal University, 355017 Stavropol, Russia; ibelasheva@ncfu.ru; 7 Department of Psychology, Saint-Petersburg State University, 199034 Saint-Petersburg, Russia; tashakhod@yandex.ru (N.K.); vikt-orija@yandex.ru (V.D.); 8 Department of Mathematics and Natural Sciences, Russian State Vocational Pedagogical University, 620143 Ekaterinburg, Russia; sarly@yandex.ru; 9 Department of Theory and Technology of Social Work, Samara National Research University, 443086 Samara, Russia; almira0913@mail.ru; 10 Department of Genetic and Clinical Psychology, Tomsk State University, 634050 Tomsk, Russia; iatamanova@yandex.ru; 11 Department of Theoretical and Practical Psychology, Kamchatka State University Named after Vitus Bering, 683032 Petropavlovsk-Kamchatskiy, Russia; anastasija81@yandex.ru (A.K.); neyaskinaju@yandex.ru (Y.N.); 12 Department of Sports Improvement, South Ural State University, 454080 Chelyabinsk, Russia; lapshin1982@yandex.ru; 13 Municipal Budgetary Educational Institution Lyceum 11, 454091 Chelyabinsk, Russia; mbmamonova@mail.ru; 14 Department of General Psychological Disciplines, Pacific State Medical University, 690002 Vladivostok, Russia; rusl-kad@yandex.ru (R.K.); iea.volkovi@mail.ru (E.V.); 15 Department of Psychology, Kemerovo State University, 650000 Kemerovo, Russia; avgrey@yahoo.com; 16 Laboratory LINP2, University of Paris Nanterre, 92001 Nanterre, France; evgeny.n.osin@gmail.com

**Keywords:** somatic burden, optimism, resilience, self-efficacy, psychological resources

## Abstract

There is scarce evidence of a relationship between positive and psychosomatic characteristics. This study aimed to examine the associations of somatic burden with psychological resources such as optimism, resilience, and general self-efficacy. Russian participants (n = 1020) completed measures of psychological resources at Time 1 and somatic symptoms at Time 2. The results showed that somatic burden decreased with greater levels of optimism, resilience, and general self-efficacy. Regarding health and sociodemographic characteristics, female sex increased somatic burden in the model with optimism scores, university education decreased somatic burden in the model with resilience scores, and history of COVID-19 disease increased somatic burden in the models with optimism, resilience, and general self-efficacy scores. This study has theoretical and practical contributions. It combines positive psychology and psychosomatic medicine and highlights the value of psychological resource interventions in the treatment and prevention of somatic burden. These findings may be useful for scientists, clinicians, and practitioners.

## 1. Introduction

Somatic symptoms are a serious threat to human well-being. Patients with somatic symptoms are often on long-term sick leave [1], suffer from anxiety and depression [2], have physical, functional, and psychological disabilities [3], and undergo numerous medical manipulations and surgical interventions [4]. They often use medical services, but over time they lose hope in medicine and visit mental health professionals [5]. General practitioners devote up to 45% of their consultations to patients with somatic symptoms, and secondary care physicians are unable to make a clear diagnosis for 50% of these patients within three months [6]. Doctors revise the diagnosis in only 8.8% of patients with functional somatic symptoms and still fear missing somatic pathology and avoid claiming the functional nature of patients’ discomfort [7]. Finally, governments in many countries incur large costs for patients with somatic symptoms because they pay for medical services and reimburse costs due to time off work and lower on-the-job productivity [8,9,10].

The link between somatic symptoms and human well-being has become even more visible during the COVID-19 pandemic. Persons with somatic symptoms felt a greater psychological burden including anxiety [11], depression [12], perceived stress [13], sense of threat [14], reduced psychological flexibility [15], and fears and ruminations about the COVID-19 pandemic [16]. The negative effects of somatic symptoms were especially severe for people with pre-existing mental and psychical disorders [17]. Persistent physical symptoms were common in 30% of patients after SARS-CoV-2 and were associated with increased risks of negative illness perception and somatic symptom disorder [18,19,20]. Some authors even suggest that Long COVID can be conceptualized as a somatic symptom disorder, because pandemic effects create a ‘perfect storm’ for persistent somatic experiences [21,22].

Harmful outcomes and the chronic discomfort of patients with somatic symptoms force scientists to search for factors preventing somatic burden. Previous studies showed that the protective factors of somatic burden included male gender [23], partner presence, greater education background, lower anxiety, depression, and co-existing medical illnesses [24], lower alexithymia [25], lower neuroticism and higher extraversion, agreeableness, conscientiousness, and openness to experience [26,27,28]. Some studies also showed that somatic symptoms were inversely associated with psychological resources, such as optimism [29,30], resilience [31,32,33], and general self-efficacy [34,35]. Considering the potential benefits of psychological resources for psychosomatic well-being and the fact that a high somatic burden was typical for a third of Russians during the COVID-19 pandemic [36], the present study aimed to examine the associations of somatic burden with optimism, resilience, and general self-efficacy in a Russian sample.

## 2. Materials and Methods

### 2.1. Procedure

Data for this study were drawn from the National Study of Somatic Burden in Russia [36]. In October–December 2021, 10,205 Russians participated in the first online survey (Time 1). One year later, in October–December 2022, we sent e-mail invitations to all participants. The response rate was 10%, resulting in a sample of 1020 participants who also completed the second online survey (Time 2). The participants were thanked for their time and received generalized feedback and recommendations.

### 2.2. Participants

Table 1 shows participant and descriptive characteristics. The participants were mostly women (78.5%) with a median age of 37 years (range 18–83 years), with a partnership status (54.2%), university education (70.7%), and history of COVID-19 disease (56.6%).

### 2.3. Instruments

The participants filled out instruments assessing personality resources (Time 1) and somatic symptoms (Time 2).

#### 2.3.1. The Life Orientation Test-Revised (LOT-R)

The LOT-R consists of 6 items measuring optimism as a tendency to expect good outcomes in various areas of life [37]. We used the Russian version of the LOT-R [38]. In this study, the total score showed good internal consistency (Cronbach’s alpha = 0.86).

#### 2.3.2. The Brief Resilience Scale (BRS)

The BRS includes 6 items assessing the perceived ability to recover from stressors [39]. We used the Russian version of the BRS [40]. In this study, the total score showed good internal consistency (Cronbach’s alpha = 0.88).

#### 2.3.3. The General Self-Efficacy Scale (GSES)

The GSES consists of 10 items measuring the general sense of perceived self-efficacy [41]. We used the Russian version of the GSES [42]. In this study, the total score showed good internal consistency (Cronbach’s alpha = 0.89).

#### 2.3.4. The Somatic Symptom Scale (SSS-8)

The SSS-8 includes 8 items assessing somatic burden through specific somatic symptoms [43]. We used the Russian version of the SSS-8 [44]. In this study, the total score showed good internal consistency (Cronbach’s alpha = 0.81).

### 2.4. Analytic Strategy

Data management and statistical analyzes were performed using R 3.1.1 12 software (R Foundation for Statistical Computing, Vienna, Austria).

A linear mixed-effects model was used to examine structure in mean somatic burden scores among four subgroups of participants with hierarchical levels of optimism, resilience, and general self-efficacy obtained by dividing the sample into quartiles (25th, 50th, 75th percentiles). The lme4 package 1.1-35.5 was used to build linear mixed-effects models and assess their quality (AIC). We used conditional R^2^ to estimate the effect size obtained from linear mixed-effects models. Conditional R^2^ was performed using the MuMln package 1.48.4. Comparisons of somatic symptoms between the obtained levels of optimism, resilience, and self-efficacy were performed using a *t*-test, correcting the result for multiple comparisons (using the Bonferroni correction).

## 3. Results

We used linear mixed-effects models (LMMs) to examine the differences in somatic burden in four subgroups of participants with hierarchical levels of optimism, resilience, and general self-efficacy. The sex, age, partnership status, educational background, and history of COVID-19 disease were entered as fixed factors, and several models were tested separately for each level of optimism, resilience, and general self-efficacy. The models were then selected using the Akaike information criterions (AICs), and the AICs were lower for models with all fixed effects. The AICs of the models are presented in Table 2.

Table 3 illustrates the results of the LMMs. Somatic burden decreased with greater levels of psychological resources including optimism, resilience, and general self-efficacy. Female sex increased somatic burden in the model with optimism scores. University education decreased somatic burden in the model with resilience scores. History of COVID-19 disease increased somatic burden in the models with optimism, resilience, and general self-efficacy scores.

**Figure 1:**
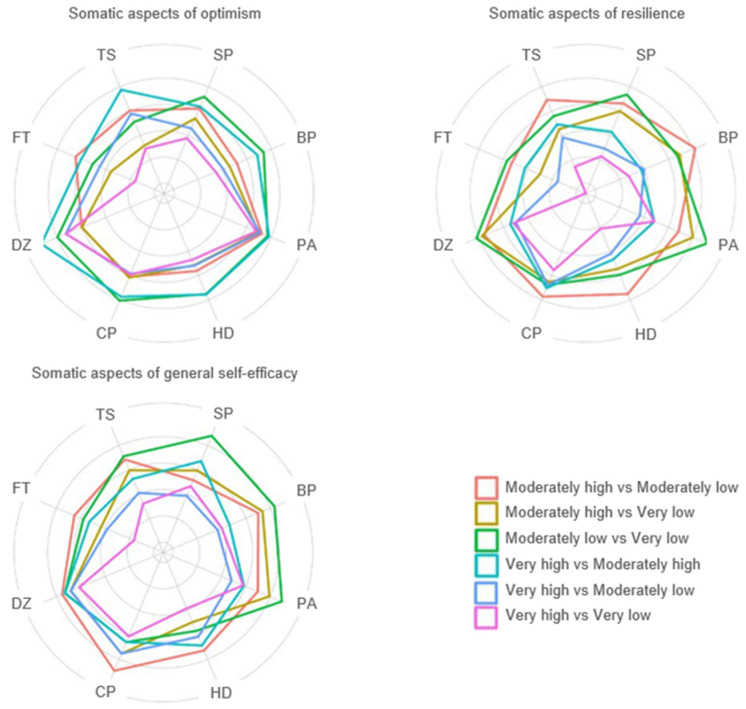
The associations of somatic burden with psychological resources. SP = stomach or bowel pain; BP = back pain; PA = pain in arms, legs, or joints; HD = headaches; CP = chest pain or shortness of breath; DZ = dizziness; FT = feeling tired or having low energy; TS = trouble sleeping. The differences between the means of the corresponding symptom scores (delta) are presented as a polygonal plot. The closer the corresponding point is to the center of the figure, the greater the delta between the selected quartiles.

Specific somatic symptoms decreased with the growth of psychological resources. Figure 1 illustrates these associations. The Supplementary Materials contains more detailed information.

## 4. Discussion

This study aimed to examine the associations of somatic burden with psychological resources. We found that persons with greater optimism, resilience, and general self-efficacy reported lower somatic burden during a one-year period, than persons with poorer optimism, resilience, and general self-efficacy.

These findings add to the body of knowledge on the associations of somatic burden with psychological resources during the COVID-19 pandemic. Previous studies showed that psychological resources protect mental health, namely, by reducing stress, anxiety, and depressive symptoms [45], loneliness [46], the risk of disordered eating behaviors [47], self-harm behaviors [48], and suicidality [49]. The protection of physical health does not seem so obvious; although, some studies revealed that psychological resources can lead to benefits for the physical health of HIV-infected patients [50], decreased pre-transplant death in lung transplant candidates [51], and increased physical functioning in cancer survivors and survivors of stem cell transplantation [52]. Regarding specific somatic symptoms, this study highlighted that most of the symptoms decreased with psychological resources. Convincing evidence for these findings has been collected in previous psychosomatic studies. Thus, optimism was inversely related to migraine and migraine-related disability [53], resilience was negatively correlated with sleep disturbances [54], and general self-efficacy was inversely associated with headache, neck pain, lower back pain, shoulder pain, upper back pain, arm pain, and pain in the feet [55].

Health and sociodemographic characteristics influenced somatic burden in different models of psychological resources. Female sex increased somatic symptoms in the model of optimism, which corresponds to the tendency of women to greater somatic burden and poorer optimism in a wide range of life expectations [23,56,57]. University education decreased somatic symptoms in the model of resilience, because more educated people are less susceptible to somatic burden and have more resources to maintain psychosomatic well-being [58,59]. Finally, a history of COVID-19 disease increased somatic symptoms in the models of optimism, resilience, and general self-efficacy, which can be associated with persistent somatic symptoms after SARS-CoV-2 infection and the negative impact of the pandemic on psychological well-being and resources [19,60,61].

This study also has some limitations, avenues, and practical implications. First, this study contained only two measurements. We believe that several tests during this period or further observations in a few years would have provided us with more data for interpretation. Second, psychological resources include not only optimism, resilience, and general self-efficacy, but also hope [62], gratitude [63], flourishing [64], positive emotions [65], character strengths [66], and positive orientations [67]. Future studies can be expanded because of these characteristics. Third, the nature of this study is self-reporting. It would be beneficial to examine the protective effects of psychological resources on somatic burden in clinical settings on patients with psychosomatic disorders and somatic symptom disorder. Despite its limitations, this study opens up the possibility of preventing somatic burden. Resilience training programs improve mental and physical health by reducing stress and anxiety symptoms [68], somatic symptoms and traumatic stress [69], negative affect and perceived stress [70], and suicidal and depressive symptoms [71]. Similar programs can alleviate or eliminate somatic burden and increase resistance to somatic and psychological distress. In addition, some exercises that increase optimism, resilience, and general self-efficacy can be used in the treatment of somatic symptom disorder and psychosomatic disorders.

## 5. Conclusions

This study highlights the role of psychological resources in reducing the burden of somatic symptoms. The theoretical contribution of our findings is the convergence of positive psychology and psychosomatic medicine. This gives researchers prospects to study the links between positive and psychosomatic characteristics. The practical contribution is to draw the attention of clinicians and practitioners to the prevention of somatic burden by improving psychological resources in counseling and psychotherapy.

## Figures and Tables

**Table 1:** Participant and descriptive characteristics.

Characteristic	Mean (SD) or n (%)
Sex, female participants, n (%)	801 (78.5)
Age (in years), mean (SD)	37.63 (12.98)
Partnership status, being in a partnership, n (%)	553 (54.2)
Educational background, university, n (%)	721 (70.7)
History of COVID-19 disease, n (%)	577 (56.6)
Optimism, mean (SD)	26.75 (7.39)
Resilience, mean (SD)	17.22 (4.89)
General self-efficacy, mean (SD)	28.41 (4.82)
Somatic burden, mean (SD)	10.15 (6.13)

**Table 2:** The AICs of the models.

Model	Optimism	Resilience	General Self-Efficacy
Model 1	6447.3	6447.3	6447.3
Model 2	6441.1	6419.2	6442.7
Model 3	6439.5	6420.7	6442.7
Model 4	6440.7	6422.0	6444.6
Model 5	6441.6	6421.8	6444.8
Model 6	6440.5	6419.0	6444.0
Model 7	6437.2	6415.9	6441.1

Fixed effects in models: Model 1 = free member; Model 2 = optimism/resilience/general self-efficacy; Model 3 = optimism/resilience/general self-efficacy + sex; Model 4 = optimism/resilience/general self-efficacy + sex + age; Model 5 = optimism/resilience/general self-efficacy + sex + age + partnership status; Model 6 = optimism/resilience/general self-efficacy + sex + age + partnership status + educational background; Model 7 = optimism/resilience/general self-efficacy + sex + age + partnership status + educational background + history of COVID-19 disease.

**Table 3:** Results of the LMMs.

Predictors	Estimates (CI)	*p*-Value
Optimism (conditional R^2^ = 0.344)		
Very low vs. Moderately low	−0.48 (−1.51; 0.54)	0.354
Very low vs. Moderately high	−1.59 (−2.61; −0.57)	0.002
Very low vs. Very high	−1.59 (−2.63; −0.55)	0.003
Female sex	0.94 (0.08; 1.81)	0.033
Age	0.02 (−0.01; 0.05)	0.150
Being in a partnership	−0.36 (−1.07; 0.36)	0.325
University education	−0.75 (−1.56; 0.05)	0.066
History of COVID-19 disease	0.82 (0.11; 1.52)	0.023
Resilience (conditional R^2^ = 0.341)		
Very low vs. Moderately low	−0.60 (−1.57; 0.37)	0.223
Very low vs. Moderately high	−0.96 (−1.96; 0.04)	0.059
Very low vs. Very high	−2.88 (−3.89; −1.87)	0.001
Female sex	0.43 (−0.43; 1.29)	0.322
Age	0.02 (−0.01; 0.05)	0.104
Being in a partnership	−0.51 (−1.21; 0.19)	0.157
University education	−0.92 (−1.72; −0.12)	0.024
History of COVID-19 disease	0.79 (0.10; 1.48)	0.025
General self-efficacy (conditional R^2^ = 0.346)		
Very low vs. Moderately low	−0.08 (−1.19; 1.02)	0.881
Very low vs. Moderately high	−0.67 (−1.63; 0.28)	0.168
Very low vs. Very high	−1.44 (−2.44; −0.45)	0.005
Female sex	0.71 (−0.16; 1.57)	0.108
Age	0.01 (−0.01; 0.04)	0.324
Being in a partnership	−0.47 (−1.18; 0.24)	0.196
University education	−0.72 (−1.52; 0.09)	0.082
History of COVID-19 disease	0.79 (0.08; 1.49)	0.028

CI = confidence interval.

## Data Availability

The data presented in this study are available on request from the corresponding author as the participants did not give consent for their raw data and transcriptions to be shared with other researchers.

## Supplementary Materials

The following supporting information can be downloaded at: https://www.mdpi.com/article/10.3390/healthcare12131338/s1, Table S1: Comparison of specific somatic symptoms within selected groups (quartiles); Table S2: Results of the correlations performed by the Pearson test between the somatic burden scores and descriptive statistics within the selected groups (quartiles); Table S3: Descriptive statistics within selected groups (quartiles).
