# Supplementary material for: Optimism, Resilience, and General Self-Efficacy Predict Lower Somatic Burden during the COVID-19 Pandemic
Source: Healthcare (Basel). 2024 Jul 5;12(13):1338. doi: 10.3390/healthcare12131338 (PMC11241252; doi:10.3390/healthcare12131338)
Supplement: Supplementary file 1 [file healthcare-12-01338-s001.zip › healthcare-3003278-supplementary.pdf]

Table S1. Comparison of specific somatic symptoms within selected groups (quartiles).

Somatic symptom	Level comparison	t-test	p-value	Bonferroni correction
Stomach or bowel pain	Moderately low vs Very low	-0.08	0.46	1
Stomach or bowel pain	Moderately high vs Very low	-0.26	0.01	1
Stomach or bowel pain	Very high vs Very low	-0.42	0	0.01
Stomach or bowel pain	Moderately high vs Moderately low	-0.18	0.09	1
Stomach or bowel pain	Very high vs Moderately low	-0.34	0	0.1
Stomach or bowel pain	Very high vs Moderately high	-0.16	0.09	1
Back pain	Moderately low vs Very low	-0.06	0.61	1
Back pain	Moderately high vs Very low	-0.34	0	0.34
Back pain	Very high vs Very low	-0.45	0	0.01
Back pain	Moderately high vs Moderately low	-0.28	0.01	1
Back pain	Very high vs Moderately low	-0.39	0	0.05
Back pain	Very high vs Moderately high	-0.11	0.26	1
Pain in arms, legs, or joints	Moderately low vs Very low	-0.03	0.81	1
Pain in arms, legs, or joints	Moderately high vs Very low	-0.1	0.37	1
Pain in arms, legs, or joints	Very high vs Very low	-0.12	0.29	1
Pain in arms, legs, or joints	Moderately high vs Moderately low	-0.08	0.49	1
Pain in arms, legs, or joints	Very high vs Moderately low	-0.09	0.4	1
Pain in arms, legs, or joints	Very high vs Moderately high	-0.02	0.88	1
Headaches	Moderately low vs Very low	-0.05	0.67	1
Headaches	Moderately high vs Very low	-0.28	0.01	0.81
Headaches	Very high vs Very low	-0.33	0	0.25
Headaches	Moderately high vs Moderately low	-0.24	0.02	1
Headaches	Very high vs Moderately low	-0.28	0.01	1
Headaches	Very high vs Moderately high	-0.04	0.65	1
Chest pain or shortness of breath	Moderately low vs Very low	0.01	0.95	1
Chest pain or shortness of breath	Moderately high vs Very low	-0.18	0.04	1
Chest pain or shortness of breath	Very high vs Very low	-0.21	0.02	1
Chest pain or shortness of breath	Moderately high vs Moderately low	-0.19	0.03	1
Chest pain or shortness of breath	Very high vs Moderately low	-0.21	0.01	1
Chest pain or shortness of breath	Very high vs Moderately high	-0.03	0.74	1
Dizziness	Moderately low vs Very low	0	0.98	1
Dizziness	Moderately high vs Very low	-0.2	0.02	1
Dizziness	Very high vs Very low	-0.07	0.41	1
Dizziness	Moderately high vs Moderately low	-0.2	0.02	1
Dizziness	Very high vs Moderately low	-0.07	0.43	1
Dizziness	Very high vs Moderately high	0.13	0.11	1
Feeling tired or having low energy	Moderately low vs Very low	-0.29	0.01	1
Feeling tired or having low energy	Moderately high vs Very low	-0.44	0	0.02
Feeling tired or having low energy	Very high vs Very low	-0.64	0	0
Feeling tired or having low energy	Moderately high vs Moderately low	-0.15	0.16	1
Feeling tired or having low energy	Very high vs Moderately low	-0.35	0	0.15
Feeling tired or having low energy	Very high vs Moderately high	-0.2	0.06	1
Trouble sleeping	Moderately low vs Very low	-0.29	0.02	1
Trouble sleeping	Moderately high vs Very low	-0.48	0	0.01
Trouble sleeping	Very high vs Very low	-0.51	0	0
Trouble sleeping	Moderately high vs Moderately low	-0.2	0.08	1
Trouble sleeping	Very high vs Moderately low	-0.22	0.05	1

Trouble sleeping	Very high vs Moderately high	-0.02	0.82	1
Stomach or bowel pain	Moderately low vs Very low	-0.06	0.55	1
Stomach or bowel pain	Moderately high vs Very low	-0.2	0.06	1
Stomach or bowel pain	Very high vs Very low	-0.57	0	0
Stomach or bowel pain	Moderately high vs Moderately low	-0.14	0.19	1
Stomach or bowel pain	Very high vs Moderately low	-0.51	0	0
Stomach or bowel pain	Very high vs Moderately high	-0.37	0	0.02
Back pain	Moderately low vs Very low	-0.13	0.26	1
Back pain	Moderately high vs Very low	-0.11	0.35	1
Back pain	Very high vs Very low	-0.53	0	0
Back pain	Moderately high vs Moderately low	0.02	0.86	1
Back pain	Very high vs Moderately low	-0.4	0	0.02
Back pain	Very high vs Moderately high	-0.42	0	0.01
Pain in arms, legs, or joints	Moderately low vs Very low	0.12	0.31	1
Pain in arms, legs, or joints	Moderately high vs Very low	0	1	1
Pain in arms, legs, or joints	Very high vs Very low	-0.32	0	0.63
Pain in arms, legs, or joints	Moderately high vs Moderately low	-0.12	0.3	1
Pain in arms, legs, or joints	Very high vs Moderately low	-0.43	0	0.01
Pain in arms, legs, or joints	Very high vs Moderately high	-0.32	0	0.56
Headaches	Moderately low vs Very low	-0.21	0.05	1
Headaches	Moderately high vs Very low	-0.25	0.02	1
Headaches	Very high vs Very low	-0.59	0	0
Headaches	Moderately high vs Moderately low	-0.05	0.64	1
Headaches	Very high vs Moderately low	-0.38	0	0.01
Headaches	Very high vs Moderately high	-0.33	0	0.15
Chest pain or shortness of breath	Moderately low vs Very low	-0.12	0.16	1
Chest pain or shortness of breath	Moderately high vs Very low	-0.15	0.11	1
Chest pain or shortness of breath	Very high vs Very low	-0.24	0.01	0.74
Chest pain or shortness of breath	Moderately high vs Moderately low	-0.03	0.76	1
Chest pain or shortness of breath	Very high vs Moderately low	-0.12	0.13	1
Chest pain or shortness of breath	Very high vs Moderately high	-0.1	0.26	1
Dizziness	Moderately low vs Very low	0.02	0.82	1
Dizziness	Moderately high vs Very low	-0.02	0.79	1
Dizziness	Very high vs Very low	-0.28	0	0.11
Dizziness	Moderately high vs Moderately low	-0.04	0.63	1
Dizziness	Very high vs Moderately low	-0.3	0	0.05
Dizziness	Very high vs Moderately high	-0.26	0	0.29
Feeling tired or having low energy	Moderately low vs Very low	-0.23	0.03	1
Feeling tired or having low energy	Moderately high vs Very low	-0.5	0	0
Feeling tired or having low energy	Very high vs Very low	-0.88	0	0
Feeling tired or having low energy	Moderately high vs Moderately low	-0.27	0.01	1
Feeling tired or having low energy	Very high vs Moderately low	-0.65	0	0
Feeling tired or having low energy	Very high vs Moderately high	-0.38	0	0.06
Trouble sleeping	Moderately low vs Very low	-0.24	0.04	1
Trouble sleeping	Moderately high vs Very low	-0.35	0	0.34
Trouble sleeping	Very high vs Very low	-0.66	0	0
Trouble sleeping	Moderately high vs Moderately low	-0.11	0.35	1
Trouble sleeping	Very high vs Moderately low	-0.42	0	0.03
Trouble sleeping	Very high vs Moderately high	-0.31	0.01	0.77
Stomach or bowel pain	Moderately low vs Very low	0.08	0.51	1
Stomach or bowel pain	Moderately high vs Very low	-0.21	0.04	1

Stomach or bowel pain	Very high vs Very low	-0.33	0	0.16
Stomach or bowel pain	Moderately high vs Moderately low	-0.28	0.01	1
Stomach or bowel pain	Very high vs Moderately low	-0.41	0	0.05
Stomach or bowel pain	Very high vs Moderately high	-0.13	0.16	1
Back pain	Moderately low vs Very low	0.03	0.78	1
Back pain	Moderately high vs Very low	-0.06	0.55	1
Back pain	Very high vs Very low	-0.4	0	0.03
Back pain	Moderately high vs Moderately low	-0.1	0.41	1
Back pain	Very high vs Moderately low	-0.43	0	0.06
Back pain	Very high vs Moderately high	-0.33	0	0.1
Pain in arms, legs, or joints	Moderately low vs Very low	0.09	0.46	1
Pain in arms, legs, or joints	Moderately high vs Very low	-0.01	0.95	1
Pain in arms, legs, or joints	Very high vs Very low	-0.22	0.04	1
Pain in arms, legs, or joints	Moderately high vs Moderately low	-0.1	0.41	1
Pain in arms, legs, or joints	Very high vs Moderately low	-0.32	0.01	1
Pain in arms, legs, or joints	Very high vs Moderately high	-0.22	0.04	1
Headaches	Moderately low vs Very low	-0.23	0.05	1
Headaches	Moderately high vs Very low	-0.3	0	0.38
Headaches	Very high vs Very low	-0.42	0	0.01
Headaches	Moderately high vs Moderately low	-0.07	0.51	1
Headaches	Very high vs Moderately low	-0.18	0.1	1
Headaches	Very high vs Moderately high	-0.11	0.23	1
Chest pain or shortness of breath	Moderately low vs Very low	-0.14	0.13	1
Chest pain or shortness of breath	Moderately high vs Very low	-0.05	0.59	1
Chest pain or shortness of breath	Very high vs Very low	-0.19	0.03	1
Chest pain or shortness of breath	Moderately high vs Moderately low	0.09	0.28	1
Chest pain or shortness of breath	Very high vs Moderately low	-0.05	0.6	1
Chest pain or shortness of breath	Very high vs Moderately high	-0.14	0.08	1
Dizziness	Moderately low vs Very low	-0.07	0.49	1
Dizziness	Moderately high vs Very low	-0.12	0.18	1
Dizziness	Very high vs Very low	-0.19	0.04	1
Dizziness	Moderately high vs Moderately low	-0.05	0.59	1
Dizziness	Very high vs Moderately low	-0.12	0.2	1
Dizziness	Very high vs Moderately high	-0.07	0.37	1
Feeling tired or having low energy	Moderately low vs Very low	-0.22	0.06	1
Feeling tired or having low energy	Moderately high vs Very low	-0.37	0	0.07
Feeling tired or having low energy	Very high vs Very low	-0.64	0	0
Feeling tired or having low energy	Moderately high vs Moderately low	-0.15	0.19	1
Feeling tired or having low energy	Very high vs Moderately low	-0.42	0	0.05
Feeling tired or having low energy	Very high vs Moderately high	-0.27	0.01	1
Trouble sleeping	Moderately low vs Very low	-0.09	0.49	1
Trouble sleeping	Moderately high vs Very low	-0.2	0.07	1
Trouble sleeping	Very high vs Very low	-0.48	0	0.01
Trouble sleeping	Moderately high vs Moderately low	-0.11	0.33	1
Trouble sleeping	Very high vs Moderately low	-0.39	0	0.21
Trouble sleeping	Very high vs Moderately high	-0.27	0.01	1

Table S2. Results of the correlations performed by the Pearson test between the somatic burden scores and descriptive statistics within the selected groups (quartiles).

Descriptive statistics	Psychological resources	Characteristic	p-value	t-estimate	Correlation
Optimism	Very low	Female sex	0.248	1.159	0.077
Optimism	Very low	Age	0.066	1.850	0.122
Optimism	Very low	Being in a partnership	0.754	0.314	0.021
Optimism	Very low	University education	0.100	-1.651	-0.109
Optimism	Very low	History of COVID-19 disease	0.080	1.756	0.116
Optimism	Moderately low	Female sex	0.143	1.470	0.094
Optimism	Moderately low	Age	0.074	1.792	0.114
Optimism	Moderately low	Being in a partnership	0.626	-0.488	-0.031
Optimism	Moderately low	University education	0.216	-1.240	-0.079
Optimism	Moderately low	History of COVID-19 disease	0.771	0.291	0.019
Optimism	Moderately high	Female sex	0.473	0.719	0.044
Optimism	Moderately high	Age	0.023	2.292	0.140
Optimism	Moderately high	Being in a partnership	0.327	0.982	0.060
Optimism	Moderately high	University education	0.647	-0.458	-0.028
Optimism	Moderately high	History of COVID-19 disease	0.008	2.662	0.162
Optimism	Very high	Female sex	0.047	1.993	0.119
Optimism	Very high	Age	0.337	0.962	0.058
Optimism	Very high	Being in a partnership	0.642	-0.466	-0.028
Optimism	Very high	University education	0.874	-0.159	-0.010
Resilience	Very high	History of COVID-19 disease	0.013	2.512	0.149
Resilience	Very low	Female sex	0.318	1.001	0.063
Resilience	Very low	Age	0.291	1.059	0.067
Resilience	Very low	Being in a partnership	0.705	-0.380	-0.024
Resilience	Very low	University education	0.101	-1.648	-0.104
Resilience	Very low	History of COVID-19 disease	0.032	2.161	0.136
Resilience	Moderately low	Female sex	0.431	-0.788	-0.049
Resilience	Moderately low	Age	0.043	2.031	0.126
Resilience	Moderately low	Being in a partnership	0.055	-1.927	-0.119
Resilience	Moderately low	University education	0.921	-0.099	-0.006
Resilience	Moderately low	History of COVID-19 disease	0.118	1.566	0.097
Resilience	Moderately high	Female sex	0.777	-0.284	-0.018
Resilience	Moderately high	Age	0.006	2.748	0.174
Resilience	Moderately high	Being in a partnership	0.524	-0.638	-0.041
Resilience	Moderately high	University education	0.224	-1.220	-0.078
Resilience	Moderately high	History of COVID-19 disease	0.053	1.942	0.124
Resilience	Very high	Female sex	0.047	1.996	0.122
Resilience	Very high	Age	0.491	0.690	0.042
Resilience	Very high	Being in a partnership	0.148	1.450	0.089
Resilience	Very high	University education	0.082	-1.747	-0.107
General self-efficacy	Very high	History of COVID-19 disease	0.373	0.892	0.055
General self-efficacy	Very low	Female sex	0.650	0.455	0.030
General self-efficacy	Very low	Age	0.109	1.607	0.104

General self-efficacy	Very low	Being in a partnership	0.895	0.133	0.009
General self-efficacy	Very low	University education	0.012	-2.541	-0.163
General self-efficacy	Very low	History of COVID-19 disease	0.132	1.513	0.098
General self-efficacy	Moderately low	Female sex	0.429	0.793	0.060
General self-efficacy	Moderately low	Age	0.255	1.141	0.087
General self-efficacy	Moderately low	Being in a partnership	0.420	-0.808	-0.062
General self-efficacy	Moderately low	University education	0.808	0.243	0.019
General self-efficacy	Moderately low	History of COVID-19 disease	0.002	3.078	0.228
General self-efficacy	Moderately high	Female sex	0.362	0.913	0.051
General self-efficacy	Moderately high	Age	0.014	2.478	0.137
General self-efficacy	Moderately high	Being in a partnership	0.632	-0.479	-0.027
General self-efficacy	Moderately high	University education	0.714	-0.366	-0.020
General self-efficacy	Moderately high	History of COVID-19 disease	0.183	1.335	0.075
General self-efficacy	Moderately low	Being in a partnership	0.420	-0.808	-0.062
General self-efficacy	Moderately low	University education	0.808	0.243	0.019
General self-efficacy	Moderately low	History of COVID-19 disease	0.002	3.078	0.228
General self-efficacy	Moderately high	Female sex	0.362	0.913	0.051
General self-efficacy	Moderately high	Age	0.014	2.478	0.137
General self-efficacy	Moderately high	Being in a partnership	0.632	-0.479	-0.027
General self-efficacy	Moderately high	University education	0.714	-0.366	-0.020
General self-efficacy	Moderately high	History of COVID-19 disease	0.183	1.335	0.075
General self-efficacy	Very high	Female sex	0.108	1.610	0.095
General self-efficacy	Very high	Age	0.844	0.197	0.012
General self-efficacy	Very high	Being in a partnership	0.880	0.151	0.009
General self-efficacy	Very high	University education	0.385	-0.870	-0.052
General self-efficacy	Very high	History of COVID-19 disease	0.181	1.340	0.079

Table S3. Descriptive statistics within selected groups (quartiles).

Descriptive statistics	Psychological resources	Age (in years), mean (SD)	Sex, female participants, n (%)	Partnership status, being in a partnership, n (%)	Educational background, university, n (%)	History of COVID-19 disease, n (%)
Optimism	Very low	34.0 (12.1)	169 (73.8)	102 (44.5)	153 (66.8)	120 (52.4)
Optimism	Moderately low	39.1 (13.7)	185 (75.2)	114 (46.3)	171 (69.5)	143 (58.1)
Optimism	Moderately high	37.5 (12.3)	216 (81.5)	157 (59.2)	194 (73.2)	156 (58.9)
Optimism	Very high	39.4 (13.1)	231 (82.5)	180 (64.3)	203 (72.5)	158 (56.4)
General self-efficacy	Very low	35.8 (13.8)	191 (79.9)	119 (49.8)	151 (63.2)	127 (53.1)
General self-efficacy	Moderately low	38.9 (13.1)	147 (84.5)	91 (52.3)	127 (73.0)	99 (56.9)
General self-efficacy	Moderately high	39.0 (12.4)	242 (75.4)	174 (54.2)	242 (75.4)	195 (60.7)
General self-efficacy	Very high	36.8 (12.6)	221 (77.3)	169 (59.1)	201 (70.3)	156 (54.5)
Resilience	Very low	35.2 (12.5)	222 (88.4)	141 (56.2)	176 (70.1)	140 (55.8)
Resilience	Moderately low	37.6 (13.5)	210 (81.1)	135 (52.1)	191 (73.7)	151 (58.3)
Resilience	Moderately high	39.6 (13.0)	183 (74.7)	130 (53.1)	174 (71.0)	141 (57.6)
Resilience	Very high	38.2 (12.7)	186 (70.2)	147 (55.5)	180 (67.9)	145 (54.7)
